# Characterization of inner capsid σA protein as a virulence factor of the pteropine orthoreovirus

**DOI:** 10.1371/journal.ppat.1014252

**Published:** 2026-05-26

**Authors:** Hayato Harima, Michihito Sasaki, Takuma Ariizumi, Nijiho Kawaguchi, Hiroko Kobayashi, Kittiya Intaruck, Takeshi Kobayashi, Takahiro Kawagishi, Yuta Kanai, Naganori Nao, Yongjin Qiu, Masahiro Kajihara, Yasuko Orba, Naoto Ito, Kanako Ishihara, Masayuki Saijo, William W. Hall, Bernard M. Hang’ombe, Hirofumi Sawa

**Affiliations:** 1 Laboratory of Veterinary Public Health, Faculty of Agriculture, Tokyo University of Agriculture and Technology, Tokyo, Japan; 2 Division of Molecular Pathobiology, International Institute for Zoonosis Control, Hokkaido University, Hokkaido, Japan; 3 Institute for Vaccine Research and Development, Hokkaido University, Hokkaido, Japan; 4 Division of International Research Promotion, International Institute for Zoonosis Control, Hokkaido University, Hokkaido, Japan; 5 Department of Virology, Research Institute for Microbial Diseases, The University of Osaka, Osaka, Japan; 6 One Health Research Center, Hokkaido University, Hokkaido, Japan; 7 Laboratory of Parasitology, Faculty of Veterinary Medicine, Hokkaido University, Hokkaido, Japan; 8 Joint Graduate School of Veterinary Sciences, Gifu University, Gifu, Japan; 9 Center for One Medicine Innovative Translational Research (COMIT), Institute for Advanced Study, Gifu University, Gifu, Japan; 10 General for Health & Welfare Bureau, Public Health Office, Sapporo, Hokkaido, Japan; 11 National Virus Reference Laboratory, School of Medicine, University College Dublin, Dublin, Ireland; 12 Global Virus Network, Tampa, Florida, United States of America; 13 Department of Para-clinical Studies, School of Veterinary and Medicine, the University of Zambia, Lusaka, Zambia; 14 Copperbelt University, Kitwe, Zambia; University of Arkansas for Medical Sciences, UNITED STATES OF AMERICA

## Abstract

Pteropine orthoreovirus (PRV) is an emerging zoonotic virus that causes pneumonia in humans. We previously isolated the PRV strain Nachunsulwe-57 (N57) from a Zambian fruit bat and demonstrated its low virulence in laboratory mice. Here, we have attempted to identify factors responsible for differences in the virulence between strain N57 and the human-derived clinical strain Miyazaki-Bali/2007 (MB). Characterization of the virulence of recombinant monoreassortant PRVs derived from highly virulent MB and low virulent N57 strains in mice revealed that compared with wild-type (WT) MB, MB-based monoreassortants carrying the L1, S1, or S2 segment from N57 exhibited attenuated virulence. Among these, the monoreassortants carrying the S1 or S2 segment exhibited reduced viral loads and reduced cytokine gene expression levels in the lungs. Genetic mapping of virulence determinants using the reciprocal monoreassortant viruses with increased virulence demonstrated that N57-based monoreassortants carrying the S1 or S2 segment of MB exhibited enhanced virulence, resulting in lower survival rate compared with WT N57. Unlike the S1 segment, the functions of the S2 segment in pathogenesis are unclear. Thus, we further investigated the functional region of the inner-capsid σA protein encoded by the S2 segment. Notably, Ser-46 of σA was identified as a key amino acid determinant of PRV virulence and is present in strains derived from humans, monkeys, and bat flies, but not those identified from bats (their natural host). Collectively, these findings demonstrate that PRV σA is one factor that regulates virulence, and that σA Ser-46 may be related to potential interspecies transmission events from bats.

## Author summary

Various pteropine orthoreovirus (PRV) strains have recently been isolated from humans and bats. Although their virological properties, including cell tropism, virus entry, and virulence, have been characterized, the mechanism by which this bat-borne virus acquires virulence in humans is unclear. Here, we identified the S1 and S2 genome segments as major determinants of PRV virulence by characterizing recombinant viruses generated using reverse genetics to exchange corresponding genomic segments between a human-derived highly virulent PRV strain and a bat-derived low virulent PRV strain. We demonstrated that the functional region of σA protein, encoded by the S2 segment, contributes to the virulence of human-derived PRV. Furthermore, cytokine profiling of PRV-infected mice suggested that infection induces an uncontrolled excessive inflammatory response, leading to death. Moreover, we found a conserved serine residue of σA in PRVs derived from many species, including humans, monkeys, and bat flies, but not in bats, suggesting an association with interspecies transmission from bats. Our findings have revealed potential mechanisms involved in highly virulent PRV strains.

## Introduction

Pteropine orthoreovirus (PRV) is an emerging bat-borne zoonotic virus responsible for acute respiratory illness in humans. The first clinical case of PRV infection was reported in Southeast Asia in 2006 [1]. Other various PRV strains were subsequently isolated from patients suffering from respiratory illness in this endemic area [2–7], with PRV antibodies detected in 1%–5% of outpatients with fever or nonspecific symptoms [8,9]. Some of these patients had contact with wild bats prior to disease onset, while others had no obvious history of bat exposure, suggesting both the occurrence of indirect transmission from bats to humans and direct human-to-human transmission [1–3,6,10].

PRV is widely distributed in bat populations and highly prevalent in Oceanian, Asian, and African countries [11–19]. It has also been isolated from mammals other than bats (e.g., monkeys) and from arthropods (e.g., blood-sucking bat flies), suggesting that other animals and arthropods may function as intermediate hosts for viral spillover [20,21]. Indeed, cynomolgus macaques experimentally infected with PRV showed active viral shedding and viral transmission to cohabiting animals via contact [22]. Thus, PRV infection is a critical public health issue in Southeast Asia that must be addressed to prevent potentially larger outbreaks and epidemics of PRV infection via bat-to-human and human-to-human transmission in Asia and other regions.

PRV belongs to the family *Spinareoviridae* and is classified into the genus *Orthoreovirus*. The genome of this segmented double-stranded RNA (dsRNA) virus comprises 10 segments (L1–L3, M1–M3, and S1–S4) encoding eight structural proteins (λA, λB, λC, μA, μB, σA, σB, and σC), four nonstructural proteins (μNS, σNS, p17, and a Fusion-associated small transmembrane [FAST] protein) [23]. The PRV virion has two capsid shells composed of inner core proteins (λA, λB, μA, and σA), the core spike protein (λC), and outer capsid proteins (μB, σB, and σC) [23,24]. A plasmid-based reverse genetics (RG) system has been established to synthesize mutated recombinant PRVs for elucidating the roles of viral proteins [25] and their functions in the PRV life cycle. For example, σC is involved in viral entry, replication, and pathogenesis [25]; FAST protein mediates intercellular fusion for efficient viral replication and virulence [26]; and p17 facilitates efficient viral replication in bat-derived cells [27].

We previously isolated the PRV strain Nachunsulwe-57 (N57) from a Zambian fruit bat and determined that it was less virulent than PRV strain Miyazaki-Bali/2007 (MB) in mice [18]. These findings prompted us to further investigate which viral proteins were responsible for differences in virulence between PRV strains. Amino acid sequence analysis with comparison of σC and FAST proteins, which are known virulence factors of PRV, between the human-derived highly virulent strain MB and the bat-derived low virulent strain N57 revealed low levels of similarity, suggesting that these differences might be related to the disparity in virulence between the two strains [18]. However, the involvement of other viral proteins cannot be exclided. Thus, this study aimed to identify viral factors associated with PRV virulence that are critical for predicting the severity of PRV infection in humans. We have identified σA (encoded by the S2 segment) as a novel virulence factor of PRV. Furthermore, Ser-46, Ser-49, and Thr-54 of σA in strain MB were revealed as essential for efficient PRV virulence.

## Results

### Generation of monoreassortant PRVs between the human-derived MB and bat-derived N57 strains

**Fig 1:**
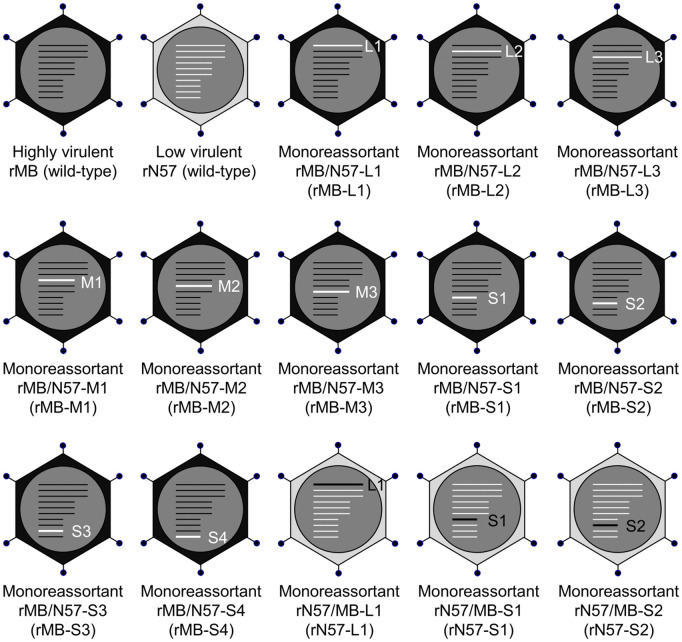
Schematic representation of monoreassortants between PRV strains MB and N57 generated in this study. MB, N57, and 13 monoreassortant viruses between PRV strains MB and N57 were recovered using RG. Recombinant strains MB and N57 were used as parent strains to generate a series of 10 monoreassortant viruses carrying each genome segment of N57 on the MB backbone for screening, and three monoreassortant viruses carrying MB-L1, MB-S1, or MB-S2 genome segments on the N57 backbone were recovered for further analysis. Genome segments derived from MB and N57 are shown in black and white, respectively. The 10 genome segments in each virion represent L1, L2, L3, M1, M2, M3, S1, S2, S2, S3, and S4 in order from top to bottom. rMB, recombinant MB; rN57, recombinant N57.

To identify PRV genome segments responsible for differences between the highly virulent MB and low virulent N57 strains, we attempted to construct monoreassortant viruses between these strains using plasmid-based RG. Recombinant wild-type (WT) MB (rMB) and N57 (rN57) were recovered from plasmids encoding the 10 genome segments of MB and N57, respectively, as previously described [25]. To obtain a panel of monoreassortant viruses carrying one genome segment of N57 within the MB strain background, each rescue plasmid of MB was replaced with the plasmid encoding the corresponding genome segment of N57 using RG. Consequently, 10 monoreassortant chimeric viruses on the MB backbone were generated as follows: rMB/N57-L1 (rMB-L1), rMB/N57-L2 (rMB-L2), rMB/N57-L3 (rMB-L3), rMB/N57-M1 (rMB-M1), rMB/N57-M2 (rMB-M2), rMB/N57-M3 (rMB-M3), rMB/N57-S1 (rMB-S1), rMB/N57-S2 (rMB-S2), rMB/N57-S3 (rMB-S3), and rMB/N57-S4 (rMB-S4) (Fig 1). We initially investigated the growth kinetics of rMB, rN57, and the 10 MB-based monoreassortant viruses in Vero E6 cells, all of which replicated well with comparable progeny virus yields at 72 hours post infection (hpi) (S1A Fig and S1 Table). These results suggest that monoreassortants between MB and N57 exert minimal effect on PRV replication in Vero E6 cells.

### Identification of genome segments responsible for the high virulence of PRV in mice

**Fig 2:**
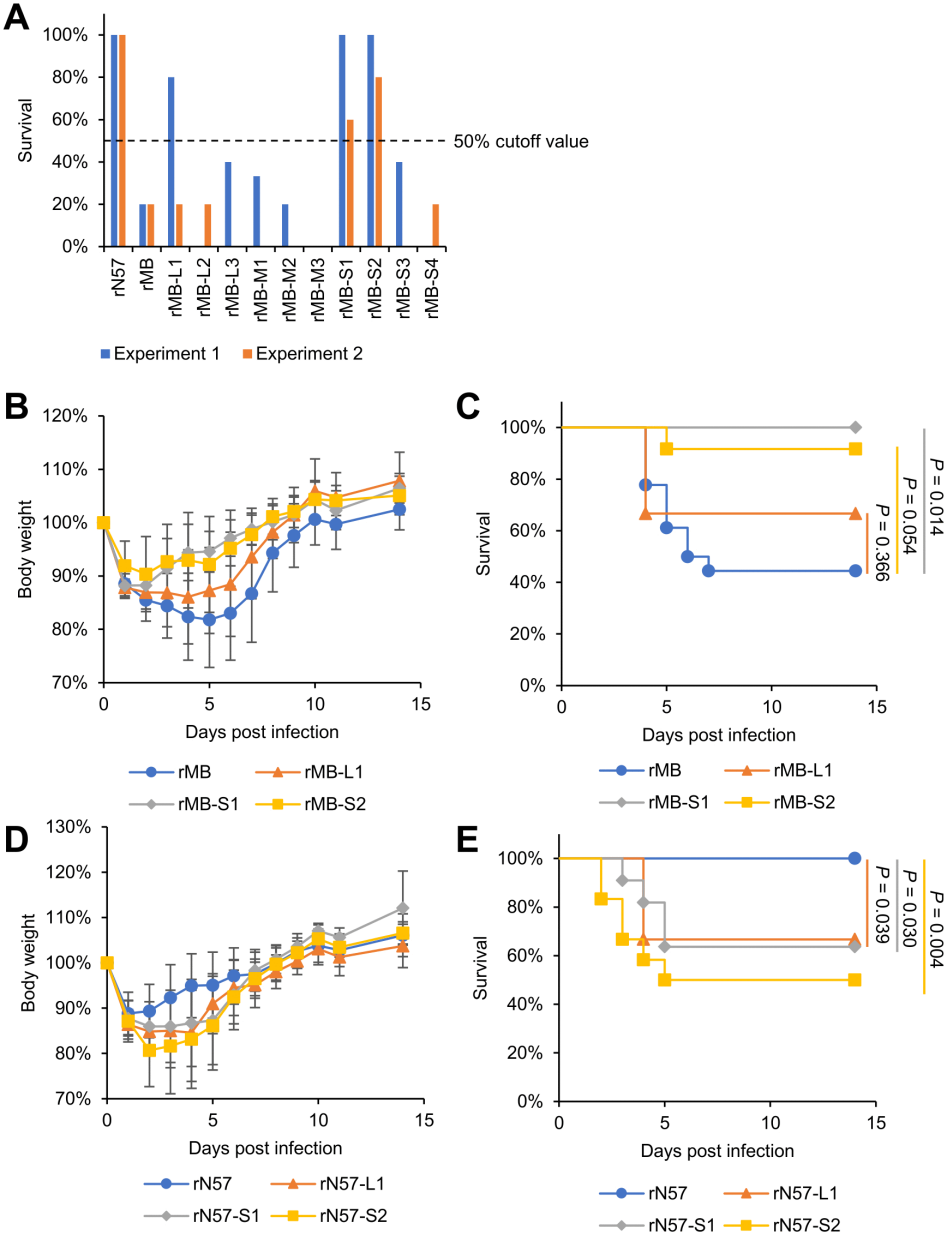
Identification of L1, S1, and S2 segments as PRV virulence factors in mice. **(A)** BALB/c mice (4 weeks old) were infected intranasally with 4 × 10^5^ PFU of rN57, rMB, rMB-L1, rMB-L2, rMB-L3, rMB-M1, rMB-M2, rMB-M3, rMB-S1, rMB-S2, rMB-S3, or rMB-S4. Body weight and survival were monitored for 14 days. Each value represents the results of two independent experiments. The dashed line indicates 50% survival as the cutoff value for screening. Details of the number of mice inoculated with each recombinant virus and their body weight are presented in supplemental S2 Table and S2 Fig, respectively. **(B–E)** BALB/c mice (4 weeks old) were infected intranasally with 4 × 10^5^ PFU of rMB (n = 18), rMB-L1 (n = 12), rMB-S1 (n = 12), or rMB-S2 (n = 12) **(B and C)** or with rN57 (n = 18), rN57-L1 (n = 12), rN57-S1 (n = 11), or rN57-S2 (n = 12) **(D and E)**. Body weight **(B and D)** and survival **(C and E)** were monitored for 14 days. Data for body weight changes represent the mean ± SD for each group. Differences in survival were analyzed using the log rank test with Holm adjustment for multiple comparisons among each of the four groups. P < 0.05, significant. rMB, recombinant MB; rN57, recombinant N57.

The virulence of the 10 MB-based monoreassortant viruses was evaluated in two independent experiments by intranasal inoculation of BALB/cCrSlc (BALB/c) mice with each monoreassortant virus (S2 Table and Figs 2A, S2A, and S2B). Consistent with a previous report [18], rMB infection resulted in a high mortality rate, whereas all mice inoculated with rN57 survived (Fig 2A). Regarding the MB-based monoreassortant viruses, > 50% of mice inoculated with rMB-S1 or rMB-S2 survived in two independent experiments (Fig 2A). Mice inoculated with rMB-L1 showed a high survival rate (80%) in Experiment 1 but a low survival rate (20%) in Experiment 2 (Fig 2A). Because the initial screening for Experiments 1 and 2 used the minimal number of animals (n = 3–5/group), we repeated the experiment using a higher number of mice infected with rMB-S1, rMB-S2 and rMB-L1 (n = 12–18/group) (Fig 2B and C). These subsequent experiments confirmed that mice inoculated with rMB-S1 or rMB-S2 experienced less body weight loss compared with mice inoculated with rMB (Fig 2B). Furthermore, the survival rate of mice inoculated with rMB-S1 or rMB-S2 was greater than that of mice inoculated with rMB (*P* = 0.014 or 0.054; Fig 2C). rMB-L1 inoculation also showed potential attenuated infection with slightly higher survival rates (*P* = 0.366; Fig 2C).

To further characterize the impact of L1, S1, and S2 segments on PRV virulence, we evaluated the virulence of the opposite monoreassortant viruses with the attenuated N57 strain background: rN57/MB-L1 (rN57-L1), rN57/MB-S1 (rN57-S1), and rN57/MB-S2 (rN57-S2) (Fig 1). First, we compared the viral growth in Vero E6 cells infected with rN57-L1, rN57-S1, rN57-S2 or WT parent viruses (rMB or rN57) to examine the effects of these monoreassortants on viral replication. The growth kinetics of rN57-L1 and rN57-S1 were similar to those of rMB and rN57 (S1B Fig and S1 Table). In contrast, the level of rN57-S2 replication was less efficient, resulting in viral titers approximately 100-fold lower than those of the WT parent viruses at 72 hpi. Next, we investigated the virulence of these N57-based monoreassortant viruses by intranasal inoculation of mice with rN57, rN57-L1, rN57-S1, or rN57-S2 and monitored their body weight and survival for 14 days (Fig 2D and 2E). The survival rate of mice inoculated with rN57-L1, rN57-S1, or rN57-S2 was significantly lower than that of mice inoculated with rN57 (*P* = 0.039, 0.030, or 0.004). This suggests that the L1, S1, and S2 genome segments are associated with the differences in virulence between strains MB and N57.

**Fig 3:**
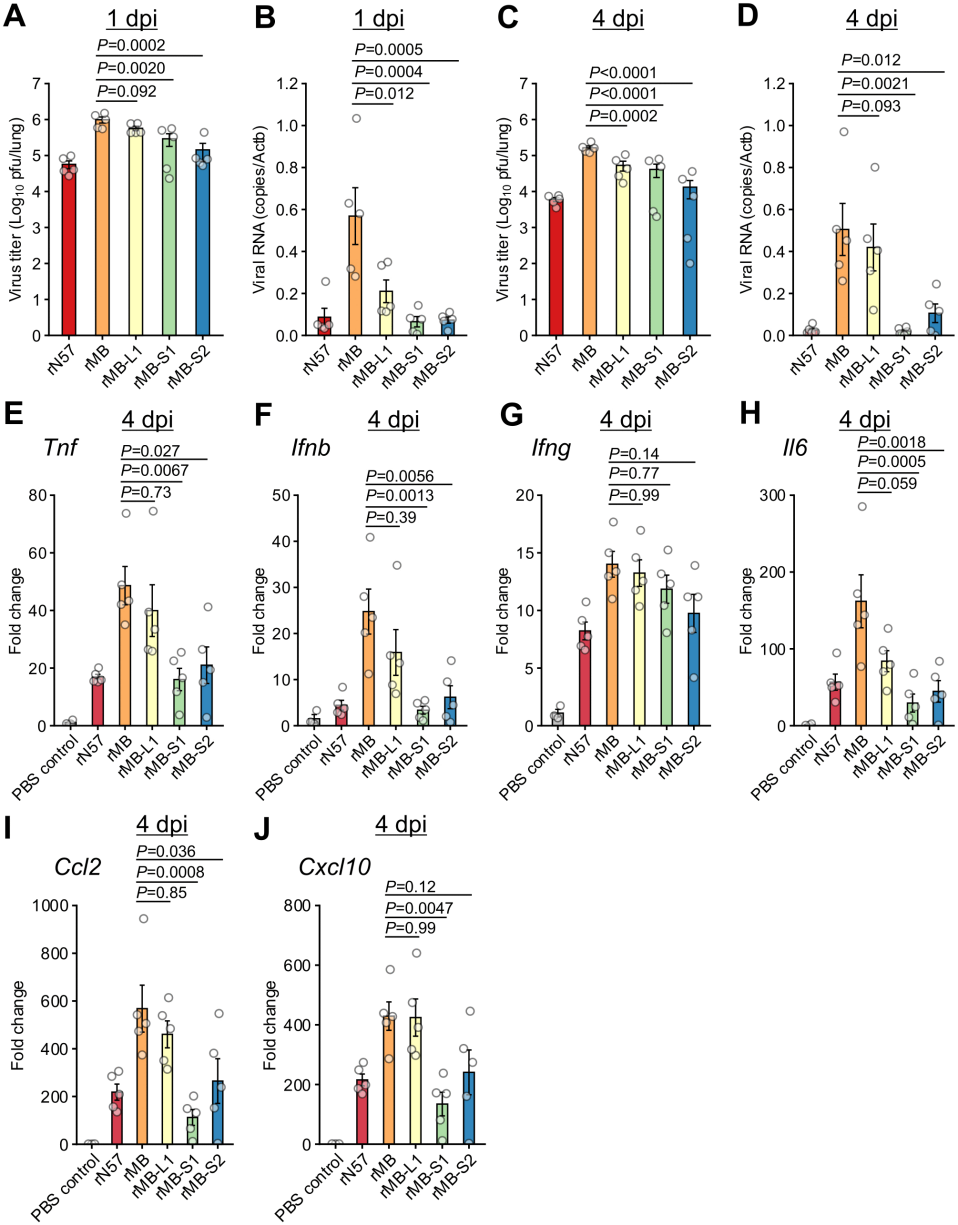
Viral load and cytokine expression profiles in lung tissue of mice infected with monoreassortant viruses. BALB/c mice (4 weeks old) were infected intranasally with 1 × 10^6^ PFU of rN57, rMB, rMB-L1, rMB-S1, or rMB-S2. **(A and C)** Viral titers in lung tissue of infected mice at 1 dpi **(A)** and 4 dpi **(C)** determined by plaque assay. **(B and D)** Viral RNA levels in lung tissue of infected mice at 1 dpi **(B)** and 4 dpi **(D)** were quantified by qRT-PCR and normalized to those of mouse β-actin (*Actb*). mRNA levels of *Tnf* **(E)**, *Ifnb* **(F)**, *Ifng* **(G)**, *Il6* **(H)**, *Ccl2* **(I)**, and *Cxcl10* **(J)** in lung tissue of infected mice at 4 dpi were quantified by qRT-PCR and normalized to those of mouse β-actin (*Actb*). Each value represents the mean ± SEM for each group (n = 5). Each dot represents an individual mouse. Statistical significance was assessed by one-way ANOVA with Tukey test. *P*-values versus rMB are indicated in the graphs. All statistical data are presented in supplemental S3 and S4 Tables. dpi, day post infection; PBS, phosphate-buffered saline; PFU, plaque-forming units; rMB, recombinant MB; rN57, recombinant N57.

To examine viral replication and inflammatory responses in the lungs of mice inoculated with the monoreassortant viruses, viral loads and cytokine gene expression levels were determined at 1 and 4 days post inoculation (dpi) (S3 and S4 Tables, Figs 3, and S3A). Mock-treated mice receiving phosphate-buffered saline (PBS) were included as a control group (Figs 3 and S3A) for the cytokine gene expression profile. Compared with the rMB inoculation group, the rMB-S1 and rMB-S2 groups showed significantly decreased virus titers and viral RNA levels in the lungs at 1 and 4 dpi (Fig 3A to 3D). In contrast, the reduction in virus titers and viral RNA levels in mice inoculated with rMB-L1 was limited at 1 and 4 dpi. At 1 dpi, the gene expression levels of cytokines (*Tnf*, *Ifnb*, *Ifng*, and *Il6*) and chemokines (*Ccl2* and *Cxcl10*) were upregulated in the lungs of mice infected with recombinant viruses compared with the PBS control group, although there was no significant difference between the rMB and MB-based monoreassortant virus inoculation groups (S3A Fig). At 4 dpi, the levels of inflammatory cytokines and chemokines in mice infected with rMB-S1 or rMB-S2 were lower than those in mice infected with rMB (Fig 3E–J). In contrast, the levels of cytokine and chemokine expression in rMB-L1 were comparable to those in mice infected with rMB. The levels of *Ifng* expression at 4 dpi in mice infected with recombinant viruses were similar among these inoculation groups (Fig 3G). Since it has been reported that *Ifng* expression is strongly upregulated in mice lungs in the late phases of PRV infection (> 5dpi) [28], analysis at 4 dpi may have been too early to detect differences in expression levels. Collectively, these observations indicate that the S1 and S2 genome segments are major determinants in the differences in the viral replication and cytokine induction in lungs between strains MB and N57.

### Identification of the region of the S2 genome segment contributing to PRV virulence in mice

**Fig 4:**
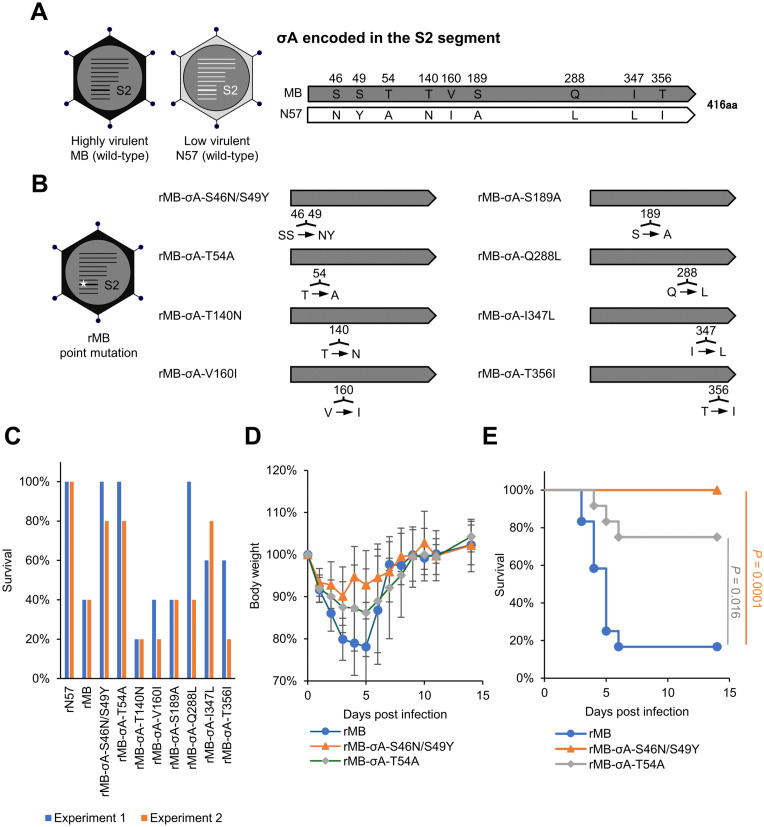
Effects of mutations in protein σA encoded by the S2 segment of PRV on mortality in mice. **(A)** Schematic diagrams showing differences between amino acid sequences in the PRV S2 segment coding for σA in strains MB and N57. The corresponding amino acids are shown with the positions numbered at the top. **(B)** Schematic diagrams showing the σA domain and recombinant viruses generated in this study. Eight recombinant viruses with single- or double-amino-acid substitutions in σA on the MB backbone were recovered by RG. **(C)** BALB/c mice (4 weeks old) were infected intranasally with 4 × 10^5^ PFU of rN57, rMB, rMB-σA-S46N/S49Y, rMB-σA-T54A, rMB-σA-T140N, rMB-σA-V160I, rMB-σA-S189A, rMB-σA-Q288L, rMB-σA-I347L, or rMB-σA-T356I. Body weight and survival were monitored for 14 days. Each value represents the results of two independent experiments. Details of the number of mice inoculated with each recombinant virus and their body weight are presented in the supplemental S5 Table and S2 Fig, respectively. **(D and E)** BALB/c mice (4 weeks old) were infected intranasally with 4 × 10^5^ PFU of rMB, rMB-σA-S46N/S49Y, or rMB-σA-T54A, and body weight **(D)** and survival **(E)** were monitored for 14 days (n = 12/group). Data for body weight changes represent the mean ± SD for each group. Differences in survival were analyzed using the log-rank test with Holm adjustment for multiple comparisons among the three groups. P < 0.05, significant. aa, amino acid; rMB, recombinant MB; rN57, recombinant N57.

The S1 genome segment of PRV encodes one structural protein (σC) and two nonstructural proteins (FAST and p17). The σC and FAST proteins are crucial for PRV pathogenesis in mice [25–27]. In contrast, the role of σA (a major inner-capsid protein encoded by the S2 genome segment) in PRV virulence is unknown. Therefore, in this study we investigated σA as a potentially new virulence factor of PRV. There are nine amino acid differences in σA proteins between strains MB and N57 (Fig 4A). To identify σA residues associated with PRV virulence, we generated recombinant viruses with an MB strain background and σA gene mutations at the sites of amino acid sequence differences between the MB and N57 strains: rMB-σA-S46N/S49Y, rMB-σA-T54A, rMB-σA-T140N, rMB-σA-V160I, rMB-σA-S189A, rMB-σA-Q288L, rMB-σA-I347L, and rMB-σA-T356I (Fig 4B). The parental WT rMB and all MB-based σA mutant viruses exhibited comparable growth kinetics in Vero E6 cells, indicating that the introduced mutations did not affect viral replication (S1C Fig and S1 Table). The virulence of MB-based σA mutant viruses was assessed using the same experimental design as for the monoreassortant viruses (Figs 2 and 3). Initial screening revealed that the rMB-σA-S46N/S49Y and rMB-σA-T54A inoculation groups had the highest survival rate (≥80%) among the parental rMB and σA mutant viruses in two independent experiments (S5 Table, Figs 4C, S2C, and S2D). Subsequent experiments confirmed that body weight changes in the rMB-σA-S46N/S49Y and rMB-σA-T54A groups were limited, and the survival rates were significantly higher than in the rMB group (Fig 4D and E). These findings demonstrated that S46N/S49Y and T54A mutations at PRV σA attenuate virulence in mice.

**Fig 5:**
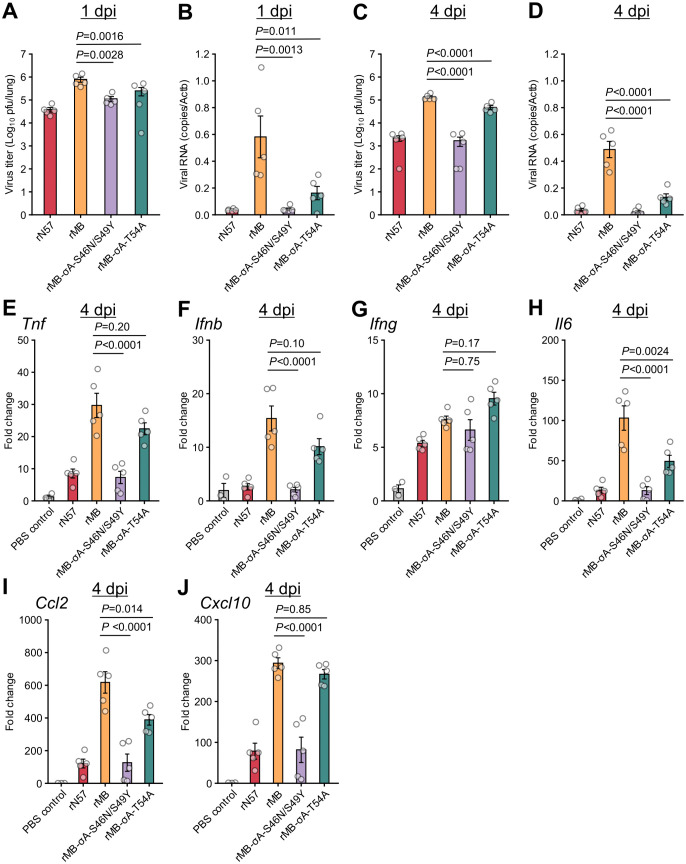
Effects of the S46N/S49Y or the T54A mutation in PRV σA on the viral replication and cytokine gene expression in lung tissues of infected mice. BALB/c mice (4 weeks old) were infected intranasally with 1 × 10^6^ PFU of rMB, rMB-σA-S46N/S49Y, or rMB-σA-T54A (n = 5/group). **(A and C)** Viral titers in lung tissue of infected mice at 1 dpi **(A)** and 4 dpi **(C)** were determined by plaque assay. **(B and D)** Viral RNA levels in lung tissue of infected mice at 1 dpi **(B)** and 4 dpi **(D)** were quantified by qRT-PCR and normalized to those of mouse β-actin (*Actb*). mRNA levels of *Tnf* **(E)**, *Ifnb* **(F),** *Ifng* **(G)**, *Il6* **(H)**, *Ccl2* **(I)**, and *Cxcl10* **(J)** in lung tissue of infected mice at 4 dpi were quantified by qRT-PCR and normalized to those of mouse β-actin (*Actb*). Each value represents the mean ± SEM for each group (n = 5). Each dot represents an individual mouse. Statistical significance was assessed by one-way ANOVA with Tukey test. P-values versus rMB are indicated in the graphs. All statistical data are detailed in supplemental S6 and S7 Tables. dpi, day post infection; PBS, phosphate-buffered saline; PFU, plaque-froming units; rMB, recombinant MB; rN57, recombinant N57.

We further characterized the attenuated virulence of rMB-σA-S46N/S49Y and rMB-σA-T54A. The virus titers and viral RNA levels in the lung tissues of the rMB-σA-S46N/S49Y and rMB-σA-T54A inoculation groups at 1 and 4 dpi were significantly lower than those of rMB group (S6 Table and Fig 5A–D). At 1 dpi, there was no significant difference in the cytokine and chemokine gene expression levels among the rMB, rMB-σA-S46N/S49Y, and rMB-σA-T54A groups (S7 Table and S3B Fig). At 4 dpi, the rMB-σA-S46N/S49Y group showed significantly lower mRNA levels for all cytokines and chemokines (except for *Ifng*) than the rMB group (Fig 5E–5J). The rMB-σA-T54A group showed significantly decreased expression levels of *Il-6* and *Ccl2* compared with the rMB group at 4dpi (Fig 5H and I) and limited reduction of *Tnf*, *Ifnb*, and *Cxcl10* expression (Fig 5E, F, and J). The *Ifng* expression levels in mice infected with σA mutant viruses at 4 dpi were similar to those in mice infected with rMB.

**Fig 6:**
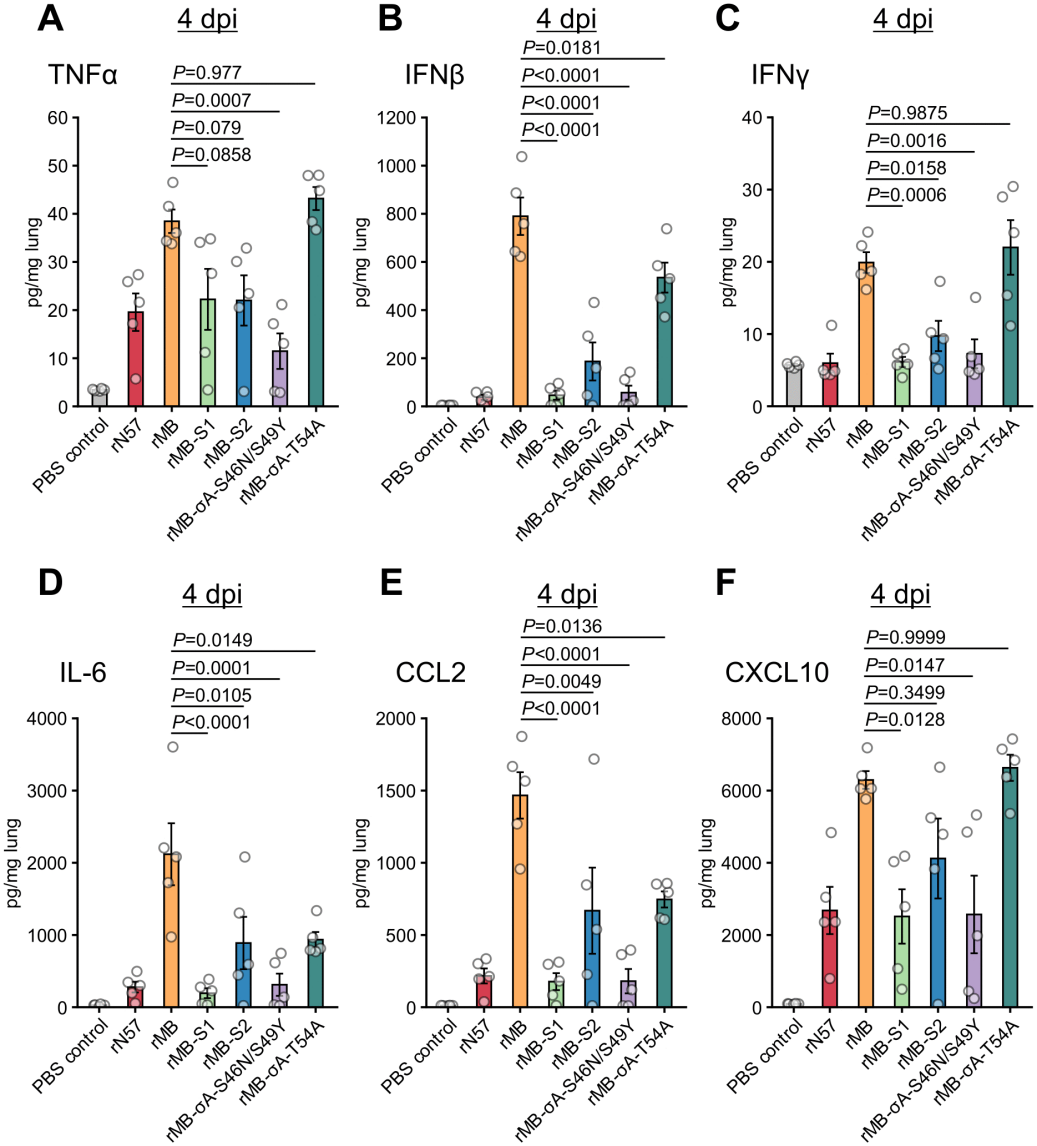
Protein expression levels of cytokines and chemokines in lung tissue of mice infected with recombinant viruses. BALB/c mice (4 weeks old) were infected intranasally with 1 × 10^6^ PFU of rN57, rMB, rMB-S1, rMB-S2, rMB-σA-S46N/S49Y, or rMB-σA-T54A. Protein levels of TNFα **(A)**, IFNβ **(B)**, IFNγ **(C)**, IL-6 **(D)**, CCL2 **(E)**, and CXCL10 **(F)** in lung tissue of infected mice at 4 dpi were measured by quantitative analyses with the MAGPIX system and normalized to the total protein concentration of lung homogenates. Each value represents the mean ± SEM for each group (n = 5). Each dot represents an individual mouse. Statistical significance was assessed by one-way ANOVA with Tukey test. *P*-values versus rMB are indicated in the graphs. All statistical data are presented in supplemental S8 Table. dpi, day post infection; PBS, phosphate-buffered saline; rMB, recombinant MB; rN57, recombinant N57.

Additionally, we investigated the protein expression levels of cytokines and chemokines in lungs of mice infected with σA mutant viruses (rMB-σA-S46N/S49Y or rMB-σA-T54A) or monoreassortant viruses (rMB-S1 or rMB-S2) at 4 dpi (S8 Table and Fig 6). Overeall, protein levels of cytokines and chemokines were largely consistent with the mRNA data, with several exceptions. Although *Ifng* mRNA levels in the lungs of mice infected with recombinant viruses were comparable among inoculation groups (Figs 3G and 5G), the rMB-S1, rMB-S2, and rMB-σA-S46N/S49Y groups showed significantly decreased IFNγ protein levels compared with the rMB group (Fig 6C). In contrast, the rMB-S1 and rMB-S2 groups showed only limited reductions in the protein expression levels of TNFα and/or CXCL10 (Fig 6A and F). Discrepancies between mRNA and protein expression levels may arise due to transcriptional and translational regulation.

**Fig 7:**
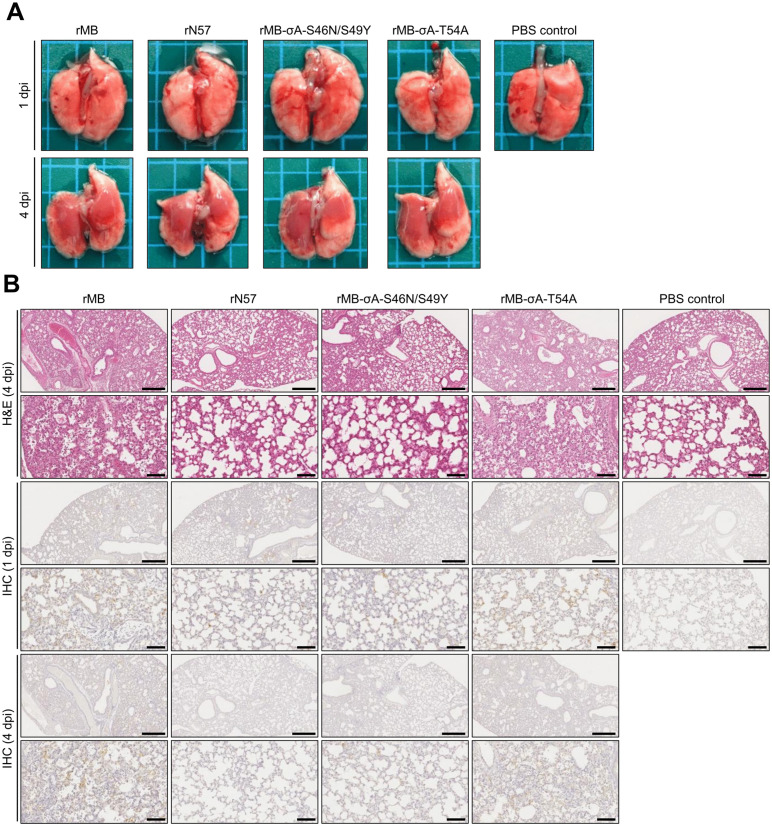
Effects of the S46N/S49Y or the T54A mutation in PRV σA on the pathological changes in lung tissue of infected mice. BALB/c mice (4 weeks old) were infected intranasally with 1 × 10^6^ PFU of rN57, rMB, rMB-σA-S46N/S49Y, or rMB-σA-T54A or with PBS (control). Lungs were harvested at 1 and 4 dpi for histological and IHC examinations. **(A)** Macroscopic images of the infected lungs. **(B)** Histopathological images of infected lung tissue following sectioning and staining with H&E and IHC staining using anti-PRV polyclonal antibody. Upper two panels, H&E staining; lower four panels, IHC staining. In each column, higher magnifications of the sections in the first, third, and fifth images (scale bars  = 500 μm) are shown in the second, fourth, and sixth images (scale bars = 100 μm), respectively. dpi, day post infection; H&E, hematoxylin and eosin; IHC, immunohistochemistry; rMB, recombinant MB; rN57, recombinant N57.

The lung tissue of mice infected with the σA mutant viruses underwent pathological examination at 1 and 4 dpi. Macroscopic examination revealed that the lungs of mice infected with rMB, rN57, rMB-σA-S46N/S49Y or rMB-σA-T54A were reddish at 4 dpi, while mild macroscopic changes were observed at 1 dpi (Fig 7A). Histopathological examination of lung tissue sections stained with hematoxylin and eosin (H&E) revealed inflammatory cell infiltration in the peribronchial, perivascular, interstitial, and alveolar space with thickening of the interstitium and the intra-alveolar septum in lung tissue of mice infected with rMB, rN57, rMB-σA-S46N/S49Y, or rMB-σA-T54A at 4 dpi (Fig 7B). In the rMB group, these histopathological changes were observed across extended areas of lungs, while the rN57 and rMB-σA-S46N/S49Y groups showed localized changes in the tissue. These changes in the rMB-σA-T54A groups were milder than those in the rMB group. Additional pathological examinations for monoreassortant rMB-S2 revealed that rN57, rMB-S2, and rMB-σA-S46N/S49Y groups showed decreased inflammation in the lungs at 4 dpi compared with the rMB group (S4 Fig). Furthermore, immunohistochemical (IHC) analyses revealed that in these inoculation groups and the rMB group, viral antigen-positive cells at 1 dpi were confined to parts of the bronchial epithelium and adjacent alveolar epithelial cells, and were observed at a slightly higher frequency than in the other group. At 4 dpi, viral antigen-positive cells were distributed across extended areas in the alveolar epithelial cells in the rMB group. In contrast, few antigen-positive cells were present in lung tissue sections of the rMB-σA-S46N/S49Y and the rN57 groups. The frequency of antigen-positive cells in the alveolar epithelial cells was lower in the rMB-σA-T54A group than the rMB group. These pathological changes correlated well with the viral RNA levels determined by reverse-transcription quantitative polymerase chain reaction (RT-qPCR) assays. Collectively, these results demonstrated that Ser-46, Ser-49, and Thr-54 of σA in PRV strain MB contribute to virulence in mice.

### Amino acid alignment and the predicted structural features of σA

**Fig 8:**
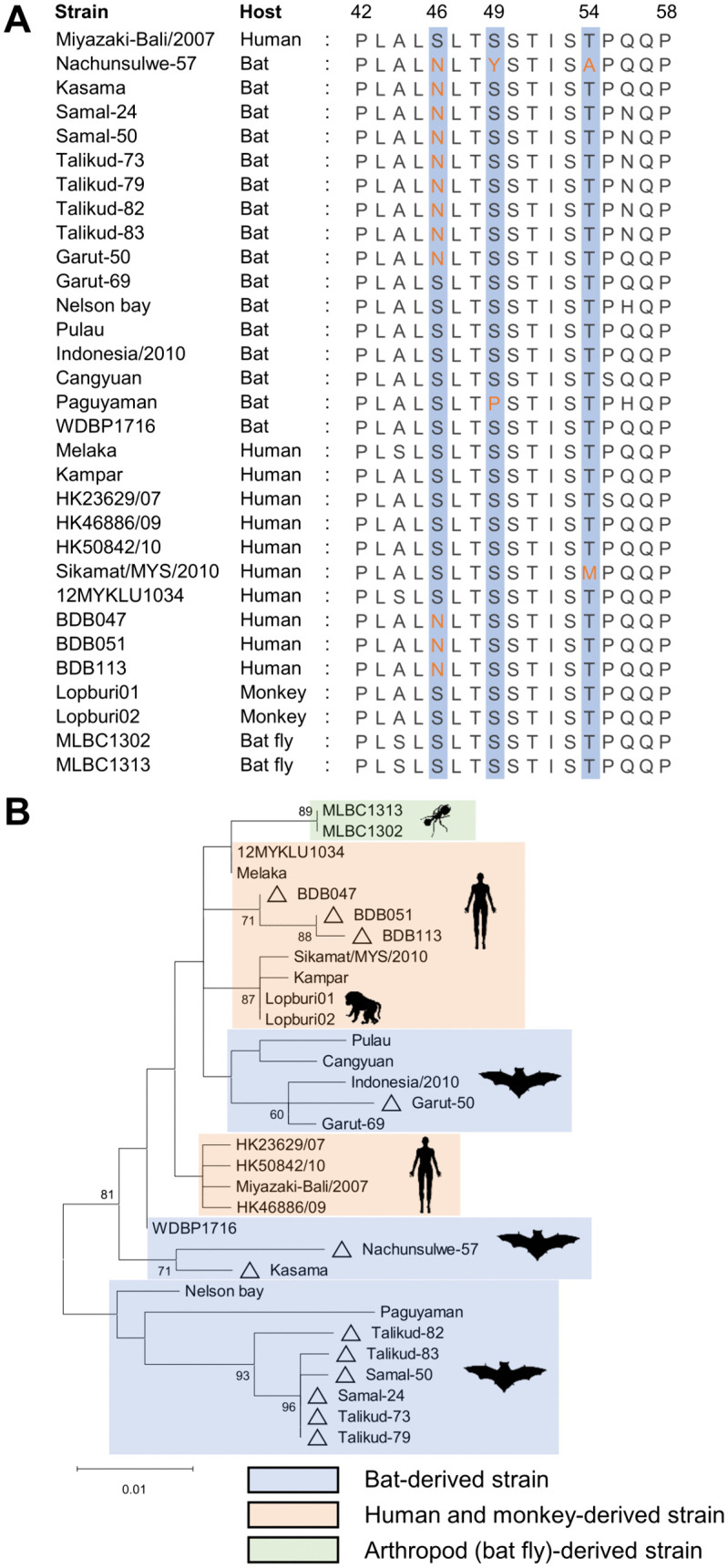
Amino acid sequence alignment and phylogenetic tree of σA constructed using various PRV strains. **(A)** Residues corresponding to positions 46, 49, and 54 of σA are shaded in blue. Amino acid mutations at the positions 46, 49, and 54 are shown in orange. **(B)** The phylogenetic tree of PRV σA was constructed using the maximum likelihood method based on the best-fit model. Bootstrap values >50% are shown on the interior branch nodes, and scale bars indicate the number of substitutions per site. Triangles represent PRV strains with σA Asn-46. Bat- and arthropod-derived PRV strains are shaded in blue and green, respectively. Human- and monkey-derived PRV strains are shaded in orange.

We investigated the conservation of identified amino acid residues among PRV strains. Ser-49 and Thr-54 of σA were highly conserved among all PRV strains except for N57, a bat-derived strain (Paguyaman), and the human-derived strain Sikamat/MYS/2010. Notably, Ser-46 of σA was not conserved in some PRV strains derived from humans and bats (Fig 8A). Phylogenetic analysis of σA sequences revealed that the bat-derived PRV strains carrying σA Asn-46 (except for Garut-50) were divided into two clusters, corresponding to African and Philippine bat lineages (Fig 8B). Interestingly, human-derived strains forming a single cluster were recently identified in patients with encephalitis during the Nipah virus outbreak in Bangladesh, and these strains harbor σA Asn-46 [29]. These findings highlight the role of Ser-46 of σA as a unique hallmark observed in a subset of bat-borne PRVs that may be potentially related to virulence.

**Fig 9:**
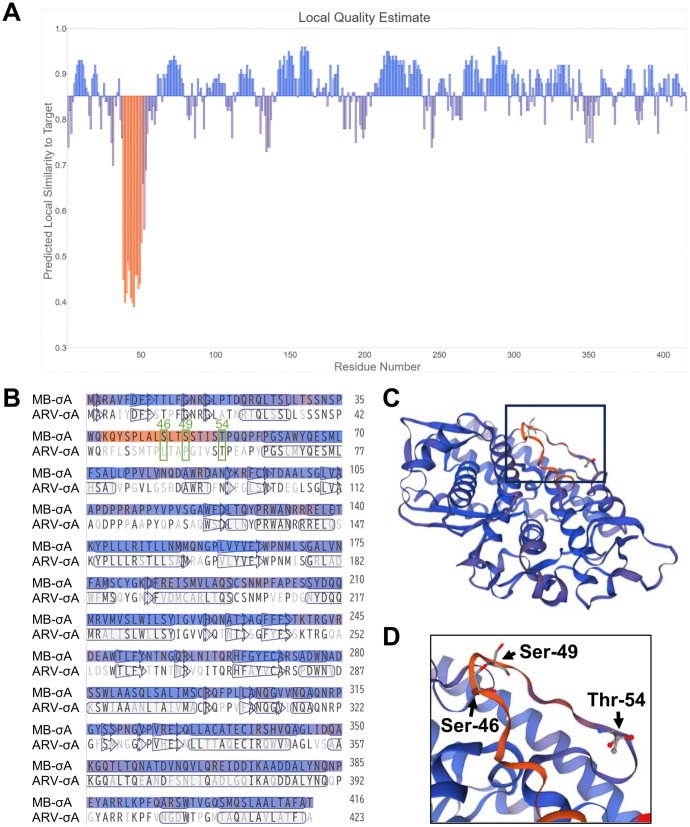
Homology modeling of MB σA using the SWISS MODEL server. **(A)** Average per-residue QMEANDisCo score for the 3D structure of MB σA. The local quality value is indicated using a color gradient from orange to blue, indicating low to high confidence. **(B)** Comparison of the amino acid sequence and the predicted secondary structures of MB σA along with the confidence levels with those of associated ARV σA. Boxes and arrows represent helices and β-strands, respectively. σA Ser-46, Ser-49, and Thr-54 in the coil are enclosed within green squares. **(C and D)** Predicted 3D structures of MB σA. **(D)** Magnified image of the boxed area in **(C)**, highlighting the loop region in which Ser-46, Ser-49, and Thr-54 are located. The per-residue QMEANDisCo scores are mapped as an orange-to-bule color gradient on the model of MB σA. ARV, avian reovirus.

The secondary structure predicted by the PSIPRED server showed the formation of a random coil structure in the MB σA region from Gln-39 to Ser-62, (i.e., including Ser-46, Ser-49, and Thr-54) (S5 Fig). Random coil structures are crucial for protein function via protein–protein interactions. Thus, S46N/S49Y and T54A mutations of MB σA may cause the dysfunction of this coil structure. Further analysis of the amino acid sequence of MB σA using the SWISS-MODEL server for comparative structure homology modeling identified a crystal structure of an avian reovirus (ARV) σA homologue (SMTL ID: 2vak.1.A, 59.62% sequence identity) as a template, and the 3D structure of MB σA was constructed based on the secondary structure predicted by the dictionary of secondary structure in proteins (DSSP) algorithm (Fig 9). The MB σA region from Leu-28 to Phe-59, including Ser-46, Ser-49, and Thr-54, formed a loop structure. Although the predicted structure showed high confidence, with a QMEANDisCo global score of 0.85 ± 0.05, the scores of MB σA residues 38–52 were <0.53. The previously solved crystal structure of ARV σA showed no interpretable electron density for residues 38–50, indicating a gap where the protein chain is disordered [30]. These results suggested the formation of a flexible loop structure in this region of MB σA and ARV σA.

## Discussion

This study used RG to identify PRV virulence factors associated with discrimination between highly virulent MB and low virulent N57 strains. The L1 and S2 genome segments were identified as responsible for the high virulence of strain MB in mice. Although the genetic mapping of virulence determinants using reciprocal monoreassortant viruses with decreased virulence suggested a limited effect of L1 monoreassortment on MB virulence, these analyses demonstrated that rN57-L1 exhibited markedly increased virulence. These findings suggested the involvement of λC and σA, encoded by the L1 and S2 segments, respectively, in PRV virulence. PRV λC is a homologue of the viral mRNA capping enzymes ARV λC and reovirus λ2. The amino-terminal 42-kDa domain (amino acids 1–385) of ARV λC and reovirus λ2 exhibits guanylyltransferase activity, and two domains (434–691 and 804–1,022) of reovirus λ2 are related to methyltransferase activities [31–33]. Additionally, an amino acid at position 636 in the methyltransferase domain was associated with IFN resistance in reoviruses [34]. It would be interesting to investigate whether there are differences in these known functions between MB and N57 strains depending on PRV λC.

PRV σA is a homologue of the dsRNA binding proteins ARV σA and reovirus σ2 [35]. In ARV σA, Arg-155 and Arg-273 are essential for dsRNA binding and nucleolar localization, and this facilitates enhanced viral replication [30,36]. Furthermore, ARV σA suppresses the host IFN response by inhibiting dsRNA-dependent protein kinase (PKR) activity [37,38]. In contrast, PRV σA Ser-46, Ser-49, and Thr-54, which are essential for PRV virulence, were located far from the dsRNA binding domains. PRV σA is a major capsid protein that also plays a crucial role in viral morphogenesis. ARV σA/reovirus σ2 stabilizes the subcore particles formed by λA/λ1 on the outer surface of the ARV λA/reovirus λ1 core-shell structure and promotes the assembly of the outer capsid proteins [39,40]. Determination of the crystal structure of the reovirus core revealed that one of three λ1-binding sites of σ2 is located at residues 39–46 which forms an ɑ-helix [40]. However, according to our PRV σA structure prediction and the ARV σA crystal structure, this region does not contain an ɑ-helix and instead formed a flexible loop structure in PRV σA and ARV σA [30]. This loop structure adopts various conformations, suggesting that the ɑ-helix of σA may become ordered upon λA binding [30]. PRV σA Ser-46, Ser-49, and Thr-54 are located within the loop structure, and λA-σA interaction may be involved in the efficient virulence of PRV. Further studies are necescary to elucidate the molecular mechanisms of virulence regulated by σA.

Although we identified multiple virulence determinants (L1, S1, and S2 genome segments) associated with the high virulence of strain MB, it remains unclear whether each determinant contributes independently to PRV virulence. It has been reported that the virulence of highly pathogenic avian influenza viruses (HPAIVs) is often regulated by polygenic factors [41–43]. For example, the compatibility between the hemagglutinin and neuraminidase is critical for the manifestation of high virulence, and multiple viral proteins act synergistically to enhance receptor binding affinity, neuraminidase activity, interferon antagonism, and viral replication, leading to lethal infection. Based on these findings in HPAIVs, it is important to consider the polygenic influences of PRV virulence determinants to better understand the molecular mechanisms underlying PRV virulence.

Amino acid sequence alignment of σA revealed that Ser-46 is conserved among known PRV strains derived from humans, simians, and bat flies, but not those derived from bats (the natural host). Furthermore, our phylogenetic analysis of σA suggests that some bat-derived and non-bat-derived PRV strains share a common ancestor possessing the σA Ser-46, with the exception of Garut-50, BDB047, BDB051, and BDB113. Collectively, these findings imply that the substitution of asparagine with serine at residue 46 of σA could have served as a driver of interspecies transmission of PRV from bats.

The RG for PRV and the mouse model of PRV infection are powerful tools that provide new insights into its pathogenicity and a valuable platform for advancing PRV research. Meanwhile, potential biosafety and biosecurity risks, including dual-use concerns, should always be carefully considered when conducting research on respiratory viruses with increased virulence, such as influenza virus [44,45]. Importantly, our genetic mapping of virulence determinants using the RG system did not generate recombinant viruses more virulent than the naturally occurring wild type strain MB. Our findings suggest that specific amino acid changes may be associated with the interspecies transmissibility of PRV from bats. Since zoonotic PRV strains have naturally emerged and circulated in bats and humans in endemic regions, the additional biosafety and biosecurity risks posed by our findings are likely limited. Furthermore, the identification of specific genetic markers may enable prediction of the virulence of emerging PRV strains and facilitate the development of antiviral agents and attenuated vaccines.

Viral infections induce inflammatory cytokine and chemokine production as part of the antiviral immune response responsible for virus elimination [46,47]. However, induction of cytokines and chemokines can also result in the presentation of different clinical symptoms, and immune system overstimulation can increase disease severity [48]. Some cytokines (*Il-6* and *Il10*) and chemokines (*Ccl2* and *Cxcl10*) are associated with COVID-19 severity [49,50]. Similarly, PRV infection in mice has been reported to upregulate the expression of various cytokines (*Tnf*, *Ifnb*, *Ifng*, and *Il6*) and chemokines (*Ccl2* and *Cxcl10*) [24,28]. To understand the relationship between cytokine/chemokine induction and PRV infection severity, we compared profiles among highly virulent and attenuated virus inoculation groups. The highly virulent rMB inoculation group showed increased expression of cytokines and chemokines in mice at 4 dpi compared with the attenuated recombinant virus inoculation group (rN57, rMB-S1, rMB-S2, or rMB-σA-S46N/S49Y), suggesting that the dysregulated and overactivated inflammatory responses caused by PRV could also contribute to lethal infections in mice, similar to those observed in COVID-19 and influenza. Thus, cytokine profiling of patients infected with PRV should be undertaken to identify biological markers of severe infection.

We successfully identified the virulence determinants of the PRV in mice, a model that has been widely used [24,51]. However, PRV infection in mice does not fully reproduce the disease phenotypes observed in humans. In fact, some PRV strains cause lethal infection in mice, whereas no fatal human cases have been reported. Differences in virulence between mouse models and humans have also been observed for other viruses. For example, an avian influenza virus isolated from patients with mild symptoms exhibited high virulence in mice, whereas other influenza viruses obtained from fatal human cases showed low virulence in mice. These findings indicate that virulence in mice does not always correlate with the disease severity in humans [52]. Therefore, the virulence phenotypes of PRV may vary among host species and might be influenced not only by viral factors but also by host factors, such as age and underlying medical conditions. To better understand the molecular mechanisms underlying PRV virulence in humans, further studies using diverse human-derived strains associated with different clinical outcomes are warranted.

In summary, we have determined key virulence factors of PRV associated with discrimination of highly virulent and low virulent strains, and the λC and σA encoded by L1 and S2 genome segments, respectively, were identified as novel virulence factors. Furthermore, we confirmed that σA Ser-46, Ser-49, and Thr-54 present in the highly virulent PRV strain MB, were necescary for efficient PRV virulence in mice. Moreover, our results suggest that Ser-46 may be associated with interspecies transmission, all of which provide novel insights into PRV virulence.

## Materials and methods

### Ethical statement

All *in vitro* and *in vivo* experiments were performed at the Biosafety Level 2 (BSL-2) facility at Tokyo University of Agriculture and Technology (approval no. Byoshitsu R4-1) and the International Institute for Zoonosis Control, Hokkaido University (approval no. 2007–020 and no. 2007–029) according to the institutional guidelines. The biosafety and containment measures of experiments using recombinant PRVs were confirmed by the Ministry of Education, Culture, Sports, Science and Technology in Japan (approved no. R2-1111 and no. R4-889) based on the national guidelines, and this study began after approval for the use of recombinant PRV on March 16, 2021. Biosafety and biosecurity practices, including personnel training and event reporting were managed by the Steering Committee for Biological Containment Facilities, International Institute for Zoonosis Control, Hokkaido University in accordance with standard operating procedures (SOPs) for BSL-2 laboratories. All experiments conducted in this study were subjected to regulatory oversight by this committee. All contaminated materials and instruments used during the experiments were decontaminated by autoclaving or treatment with sodium hypochlorite in accordance with SOPs, and all pathogenic biological materials were inactivated upon completion of the research. All animal experiments were performed according to the National University Corporation, Hokkaido University Regulations on Animal Experimentation, and the study protocol was approved by the Institutional Animal Care and Use Committee of Hokkaido University (approval no. 20–0026 and no. 25–0045). Overall, this article was in accordance with the minimum information about a high containment laboratory experiment (MIHCLE) reporting standard developed for research with high-risk pathogens [53].

### Cells and viruses

African green monkey kidney (Vero E6) cells (ATCC CRL-1586) and human embryonic kidney-derived 293T (HEK293T) cells (Lenti-X 293T cell line; Takara code 632180) were obtained from the American Type Culture Collection and Takara Bio Inc., respectively. Vero E6 and HEK293T cells were maintained in Dulbecco’s modified Eagle’s medium (DMEM) supplemented with 10% fetal bovine serum (FBS) and penicillin–streptomycin (P/S) at 37°C with 5% CO_2_. Baby hamster kidney cells stably expressing T7 RNA polymerase (BHK/T7-9) were established as described previously [54]. BHK/T7-9 cells were maintained in minimum essential medium supplemented with 10% FBS and P/S at 37°C with 5% CO_2_. The PRV strain N57 was isolated from an Egyptian fruit bat captured in Zambia as described previously [18]. Briefly, collected bat tissues were transported to our BSL-2 laboratory in accordance with International Air Transport Association (IATA) guidelines and stored at –80°C in the laboratory. For virus isolation, a PRV-positive bat colon was inoculated with Vero E6 cells, and PRV was identified in the cells showing a clear cytopathic effect (CPE) with syncytium formation by next generation sequencing. The isolated PRV strain N57 was stored at –80°C in a BSL-2 laboratory in accordance with the institutional biosafety guidelines.

### Plasmids

pT7-L1MB, pT7-L2MB, pT7-L3MB, pT7-M1MB, pT7-M2MB, pT7-M3MB, pT7-S1MB, pT7-S2MB, pT7-S3MB, and pT7-S4MB plasmids encoding the full-length cDNA of each genome segment (L1, L2, L3, M1, M2, M3, S1, S2, S3, and S4, respectively) for RG of PRV strain MB have been constructed as described previously [25]. To generate rescue plasmids containing viral cDNA of each N57 genome segment flanked by the T7 promoter and the hepatitis delta virus (HDV) ribozyme sequences (pT7-L1N57, pT7-L2N57, pT7-L3N57, pT7-M1N57, pT7-M2N57, pT7-M3N57, pT7-S1N57, pT7-S2N57, pT7-S3N57, and pT7-S4N57), the full-length cDNAs of each genome segment of strain N57 were amplified by RT-PCR, and cloned into plasmid pT7-S1MB by replacing the S1 sequence derived from strain MB using an In-Fusion HD Cloning Kit (Takara). To generate rescue plasmids for the σA mutant viruses on the MB backbone, pT7-S2MB was altered by the site-directed mutagenesis using a PrimeSTAR Mutagenesis Basal Kit (Takara), resulting in recombinants with single or double amino acid substitutions in σA between strains MB and N57 (pT7-S2MB-σA-S46N/S49Y, pT7-S2MB-σA-T54A, pT7-S2MB-σA-T140N, pT7-S2MB-σA-V160I, pT7-S2MB-σA-S189A, pT7-S2MB-σA-Q288L, pT7-S2MB-σA-I347L, and pT7-S2MB-σA-T356I). S9 Table presents the primers used for plasmid construction.

### Synthesis and concentration of recombinant viruses

Recombinant viruses were generated using plasmid-based RGs with BHK/T7-9 cells as described previously [25]. Briefly, BHK-T7-9 cells in 6-well plates (1–4.5 × 10^5^ cells/well) were co-transfected with 10 plasmids encoding the full-length cDNA of each genome segment using 2–5 μl of TransIT-LT1 transfection reagent (Takara) per 1 μg of plasmid DNA, according to the manufacturer’s instructions. The amount of each plasmid was as follows: plasmids encoding L1, L2, or L3 genome segments, 0.66 μg each; plasmids encoding M1, M2, or M3 genome segments, 0.58 μg each; plasmids encoding S1, S2, S3, and S4 genome segments, 0.5 μg each. At 2–4 days post-transfection, the supernatant from cells lysed by a freeze-thaw cycle was inoculated onto monolayers of Vero E6 cells to confirm the recovery of recombinant viruses. WT rMB and rN57 strains, a series of monoreassortant viruses carrying each genome segment of N57 on the MB backbone, and three monoreassortant viruses carrying MB-L1, MB-S1, or MB-S2 genome segments on the N57 backbone were generated by RG (Fig 1). To generate MB-based recombinant viruses in which the σA gene mutations were introduced into different amino acid sequences between MB and N57 strains, the pT7-S2MB plasmid was replaced by plasmids with σA mutations (Fig 4B). The reassortments and σA mutations from recombinant viruses were confirmed by Sanger sequencing of the viral genome extracted from recovered viruses. Propagation of recovered viruses was performed as described previously [18]. Briefly, recombinant viruses were propagated in Vero E6 cells in DMEM supplemented with 2% FBS for growth kinetics analysis. For experimental infection of mice, recombinant viruses were propagated in HEK293T cells in 150-mm dishes in DMEM supplemented with 5–10% FBS, and harvested cells were lysed by a freeze-thaw cycle. After removal of cell debris by centrifugation, the supernatant (approximately 30 ml) was layered onto 5 ml of 20% sucrose in PBS and ultracentrifuged at 174,000 × *g* and 4°C for 2 hours. The concentrated viruses were resuspended in 1 ml of PBS. The constructed recombinant viruses were stored at –80°C in a BSL-2 laboratory in accordance with the institutional biosafety guidelines.

### Measurement of virus titers

Infectious virus titers were determined by plaque assay using Vero E6 cells as described previously [18]. Briefly, confluent monolayers of Vero E6 cells were infected with 10-fold serial dilution of recombinant viruses. After virus adsorption, infected cells monolayers were overlaid with DMEM supplemented with 2% FBS and 0.7% agarose. Plaque-forming units (PFU) were determined at 2 or 3 dpi. For growth kinetics analysis, Vero E6 cells were infected with recombinant viruses at a multiplicity of infection (MOI) of 0.01. After adsorption for 1 hour, the cells were washed twice with DMEM and incubated in DMEM supplemented with 2% FBS. Culture supernatants were collected at 6, 24, 48, and 72 hpi, and infectious virus titers were determined by plaque assay using Vero E6 cells.

### Animal studies

Specific-pathogen free BALB/c mice (4 weeks old; Japan SLC Inc.) were intranasally inoculated with 4 × 10^5^ or 1 × 10^6^ PFU of each of the indicated recombinant viruses in 40 μl of PBS under isoflurane anesthesia. The body weight of each mouse was monitored for 14 days, and a weight loss >25% of the initial body weight was defined as a humane endpoint in the PRV infection model as previously described [51]. For measurement of viral load, cytokine gene expression, cytokine protein expression (n = 5/group) and histopathological examinations (n = 3/group), lung tissues were harvested from mice infected with recombinants viruses at 1 or 4 dpi and control mice (PBS only).

### Measurement of viral load and cytokine gene expression in lungs of infected mice by plaque assay and RT-qPCR assay

The lungs of mice infected with recombinant viruses were homogenized in 2 ml of PBS using a TissueRuptor (Qiagen). To determine the infectious viral titers in lungs, a portion of the homogenate was subjected to plaque assay using Vero E6 cells as described in the “Measurement of virus titers” section of Materials and Method. Another portion of the homogenate was used to measure viral RNA and cytokine gene expression levels. Briefly, total RNA was extracted from the homogenate using a PureLink RNA Mini Kit (Invitrogen). The extracted RNA was used as the template for RT-qPCR using One Step PrimeScript III RT-qPCR Mix, with UNG (Takara) on a QuantStudio 7 Flex Real-time PCR system (Applied Biosystems). Primers (N57MB-L1-qPCR-F and N57-L1-qPCR-R) and a probe (N57-L1-P) targeting the PRV L1 genome segment of strain N57 were designed and synthesized by FASMAC Co., Ltd. (S9 Table). N57-L1-qPCR-R and N57-L1-P were modified to detect strain MB, and a reverse primer (MB-L1-qPCR-R) and probe (MB-L1-P) were synthesized by Integrated DNA Technologies Inc. (S9 Table). Other primers and probes used to detect mouse β-actin (*Actb*), *Tnf*, *Ifnb*, *Ifng*, *Il6*, *Ccl2*, and *Cxcl10* were described previously [28]. Viral RNA and cytokine gene expression levels were normalized to *Actb*, and the relative gene expression level was calculated using the 2^−ΔCt^ and 2^−ΔΔCt^ methods, respectively.

### Measurement of protein levels of cytokines and chemokines in lungs of infected mice

Lung homogenates were centrifuged at 17,000 × *g* and 4°C for 5 min to remove the tissue aggregates and the supernatants were diluted 5-fold with PBS containing a protease inhibitor cocktail (cOmplete ULTRA tablet, Roche). Protein concentrations of the diluted samples were determined by the Pierce 660 nm Protein Assay (Thermo Fisher Scientific). The diluted samples derived from lung homogenates were used to determine the protein level of TNFα, IFNβ, IFNγ, IL-6, CCL2, and CXCL10 using the MILLIPLEX Mouse Cytokine/Chemokine/GF Expanded Panel 1 (Merck) and the MAGPIX system (Luminex) according to the manufacturer’s instructions. The limit of detection (LOD) for the assay was as follows: TNFα, 3.66 pg/mL; IFNβ, 4.88 pg/mL; IFNγ, 6.10 pg/mL; IL-6, 7.32 pg/mL; CCL2, 12.21 pg/mL; CXCL10, 8.54 pg/mL. Samples below the LOD were given values at the LOD for statistical analysis. Obtained protein levels of cytokines and chemokines in the diluted samples were normalized to total protein concentration and expressed as pg/mg total protein of lungs.

### Histopathological and IHC assays

The lungs of mice infected with recombinant viruses underwent pathological examination as described previously [18]. Lung tissue sections were stained with H&E for histopathological evaluation. For IHC analysis, tissue sections were pretreated with 0.3% H_2_O_2_ in methanol and incubated with a polyclonal guinea pig anti-PRV serum. After washing with PBS containing tween 20, viral antigen was detected using the VECTASTAIN ABC HRP Kit (guinea pig IgG; Vector Laboratories) and the Histofine diamino benzidine substrate (Nichirei) according to the manufacturer’s instructions. The sections were counterstained with hematoxylin for nuclear staining. The slides were scanned at 40 x magnification using a NanoZoomer S60v2 digital slide scanner (Hamamatsu Photonics), and histopathological images were acquired using NDP.view2 software (Hamamatsu Photonics).

### Bioinformatics analysis

Multiple alignment of the amino acid sequences of σA protein was performed using MEGA X software with the MUSCLE tool (S10 Table) [55]. The resulting data were used to construct a phylogenetic tree using the maximum likelihood method based on the JTT + G model with 1000 bootstrap replicates. The secondary structure of the σA protein was predicted by the PSIPRED 4.0 web server (https://bioinf.cs.ucl.ac.uk/psipred/, accessed on June 4, 2025). Homology models were constructed using the SWISS-MODEL homology modeling server (https://swissmodel.expasy.org/, accessed on June 4, 2025) [56–58].

### Statistical analysis

Statistical analyses were performed using R v4.2.2 software (The R Foundation for Statistical Computing) with EZR v1.61 (Jichi Medical University), a graphical user interface for R [59]. The survival curves of infected mice were analyzed using the log-rank test with Holm adjustment for multiple comparisons. Differences in viral titers, relative viral RNA levels, relative cytokine gene expression levels, and cytokine protein expression levels in lung tissue or Vero E6 cells were analyzed by one-way ANOVA followed by Tukey test. *P*-values < 0.05 were considered statistically significant.

## Supporting information

S1 Table Results of one-way ANOVA with Tukey test for differences in viral titers in Vero E6 cells infected with recombinant viruses at 72 hours post infection. (XLSX)

S2 Table Virulence of monoreassortant viruses in mice. (XLSX)

S3 Table Results of one-way ANOVA with Tukey test for differences in viral loads of mice lungs infected with monoreassortant viruses. (XLSX)

S4 Table Results of one-way ANOVA with Tukey test for differences in mRNA levels of cytokine gene expression of mice lungs infected with monoreassortant viruses. (XLSX)

S5 Table Virulence of recombinant viruses with various mutations in σA in mice. (XLSX)

S6 Table Results of one-way ANOVA with Tukey test for differences in viral loads of mice lungs infected with recombinant viruses with S46N/S49Y or T54A mutation in σA. (XLSX)

S7 Table Results of one-way ANOVA with Tukey test for differences in mRNA levels of cytokine gene expression of mice lungs infected with recombinant viruses with S46N/S49Y or T54A mutation in σA. (XLSX)

S8 Table Results of one-way ANOVA with Tukey test for differences in protein levels of cytokines and chemokines in lungs infected with recombinant viruses at 4 days post infection. (XLSX)

S9 Table Primers and probes used in this study. (XLSX)

S10 Table PRV strains used for amino acid sequence alignment of σA. (XLSX)

S1 Fig Growth kinetics of generated recombinant viruses in cell culture. (A to C) Vero E6 cells were infected with rN57 (A to C), rMB (A to C), rMB-L1 (A), rMB-L2 (A), rMB-L3 (A), rMB-M1 (A), rMB-M2 (A), rMB-M3 (A), rMB-S1 (A), rMB-S2 (A), rMB-S3 (A), rMB-S4 (A), rN57-L1 (B), rN57-S1 (B), rN57-S2 (B), rMB-σA-S46N/S49Y (C), rMB-σA-T54A (C), rMB-σA-T140N (C), rMB-σA-V160I (C), rMB-σA-S189A (C), rMB-σA-Q288L (C), rMB-σA-I347L (C), and rMB-σA-T356I (C) at a multiplicity of infection of 0.01. The supernatants were harvested at the indicated times, and viral titers were determined by plaque assay. Each value represents the mean ± SEM of the results of three independent experiments. hpi, hour post infection; PFU, plaque-forming units; rMB, recombinant MB; rN57, recombinant N57.
(TIF)

S2 Fig Body weight changes in mice infected with recombinant PRVs. (A and B) Body weight changes in mice infected intranasally with 4 × 10^5^ PFU of rN57, rMB, rMB-L1, rMB-L2, rMB-L3, rMB-M1, rMB-M2, rMB-M3, rMB-S1, rMB-S2, rMB-S3, or rMB-S4. The results of two independent experiments are shown in Experiment 1 (A) and Experiment 2 (B). (C and D) Body weight changes in mice infected intranasally with 4 × 10^5^ PFU of rN57, rMB, rMB-σA-S46N/S49Y, rMB-σA-T54A, rMB-σA-T140N, rMB-σA-V160I, rMB-σA-S189A, rMB-σA-Q288L, rMB-σA-I347L, or rMB-σA-T356I. The results of two independent experiments are shown as Experiment 1 (C) and Experiment 2 (D). Data represent the mean ± SD for each group. rMB, recombinant MB; rN57, recombinant N57.
(TIF)

S3 Fig Cytokine gene expression in lung tissue of mice infected with recombinant PRVs at 1 dpi. (A) mRNA levels of *Tnf*, *Ifnb*, *Ifng*, *Il6*, *Ccl2*, and *Cxcl10* in lung tissue of mice infected with rN57, rMB, rMB-L1, rMB-S1, or rMB-S2 at 1 dpi. (B) mRNA levels of *Tnf*, *Ifnb*, *Ifng*, *Il6*, *Ccl2*, and *Cxcl10* in lung tissue of mice infected with rN57, rMB, rMB-σA-S46N/S49Y, or rMB-σA-T54A at 1 dpi. Data were normalized to mouse β-actin (*Actb*). Each value represents the mean ± SEM for each group (n = 5). Each dot represents an individual mouse. dpi, days post infection; PBS, phosphate-buffered saline; rMB, recombinant MB; rN57, recombinant N57.
(TIF)

S4 Fig Pathological changes in lung tissue of infected mice infected with monoreassortant and σA mutant virus. BALB/c mice (4 weeks old) were infected intranasally with 1 × 10^6^ PFU of rN57, rMB, rMB-S2, or rMB-σA-S46N/S49Y or with PBS (control). Lungs were harvested at 4 dpi for histological examination. (A) Macroscopic images of the infected lungs. (B) Histopathological images of infected lung tissue following sectioning and H&E staining. Higher magnifications of the sections in the first images (scale bars = 500 μm) are shown in the second images (scale bars = 100 μm). dpi, day post infection; H&E, hematoxylin and eosin; rMB, recombinant MB; rN57, recombinant N57.
(TIF)

S5 Fig Predicted secondary structure of MB σA using the PSIPRED server. Pink, helices; yellow, β strands; gray, coils. The positions of σA Ser-46, Ser-49, and Thr-54 in the coil are highlighted within green squares.
(TIF)
