# Supplementary figures and images for: Characterization of inner capsid σA protein as a virulence factor of the pteropine orthoreovirus
Source: PLoS Pathog. 2026 May 26;22(5):e1014252. doi: 10.1371/journal.ppat.1014252 (PMC13225656; doi:10.1371/journal.ppat.1014252)

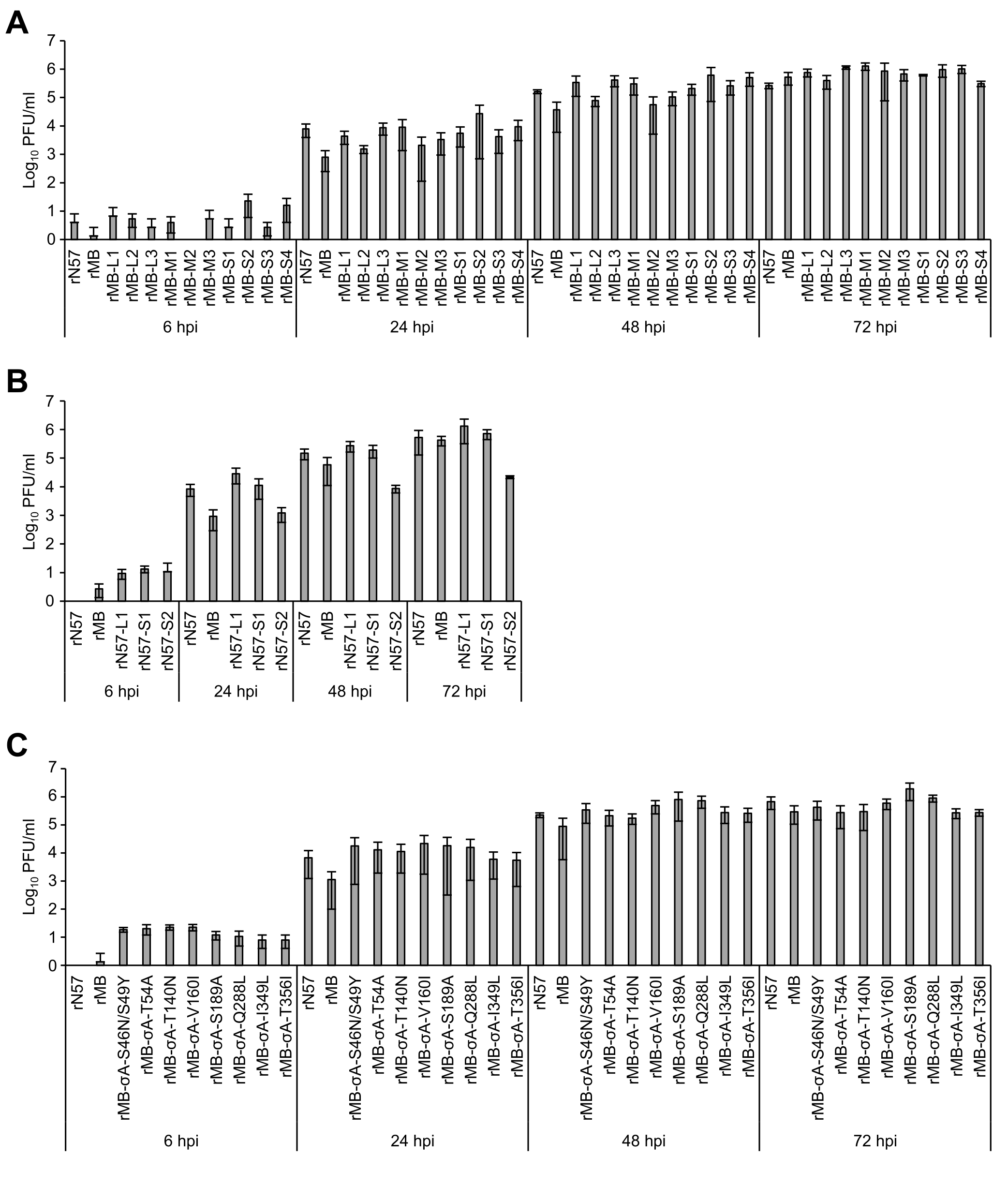

Supplement: S1 Fig — (A to C) Vero E6 cells were infected with rN57 (A to C), rMB (A to C), rMB-L1 (A), rMB-L2 (A), rMB-L3 (A), rMB-M1 (A), rMB-M2 (A), rMB-M3 (A), rMB-S1 (A), rMB-S2 (A), rMB-S3 (A), rMB-S4 (A), rN57-L1 (B), rN57-S1 (B), rN57-S2 (B), rMB-σA-S46N/S49Y (C), rMB-σA-T54A (C), rMB-σA-T140N (C), rMB-σA-V160I (C), rMB-σA-S189A (C), rMB-σA-Q288L (C), rMB-σA-I347L (C), and rMB-σA-T356I (C) at a multiplicity of infection of 0.01. The supernatants were harvested at the indicated times, and viral titers were determined by plaque assay. Each value represents the mean ± SEM of the results of three independent experiments. hpi, hour post infection; PFU, plaque-forming units; rMB, recombinant MB; rN57, recombinant N57. (TIF) [file ppat.1014252.s011.tif]

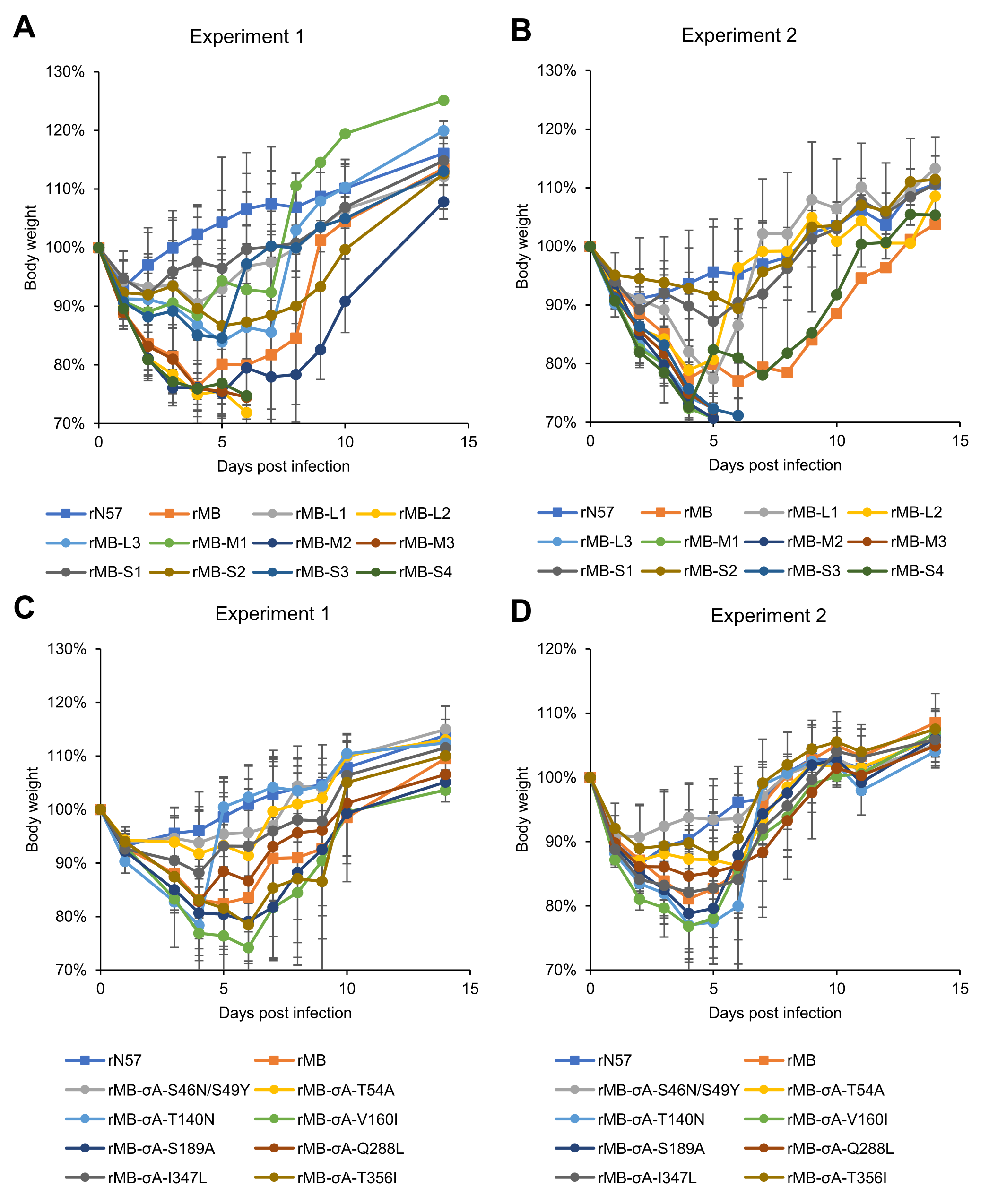

Supplement: S2 Fig — (A and B) Body weight changes in mice infected intranasally with 4 × 10^5^ PFU of rN57, rMB, rMB-L1, rMB-L2, rMB-L3, rMB-M1, rMB-M2, rMB-M3, rMB-S1, rMB-S2, rMB-S3, or rMB-S4. The results of two independent experiments are shown in Experiment 1 (A) and Experiment 2 (B). (C and D) Body weight changes in mice infected intranasally with 4 × 10^5^ PFU of rN57, rMB, rMB-σA-S46N/S49Y, rMB-σA-T54A, rMB-σA-T140N, rMB-σA-V160I, rMB-σA-S189A, rMB-σA-Q288L, rMB-σA-I347L, or rMB-σA-T356I. The results of two independent experiments are shown as Experiment 1 (C) and Experiment 2 (D). Data represent the mean ± SD for each group. rMB, recombinant MB; rN57, recombinant N57. (TIF) [file ppat.1014252.s012.tif]

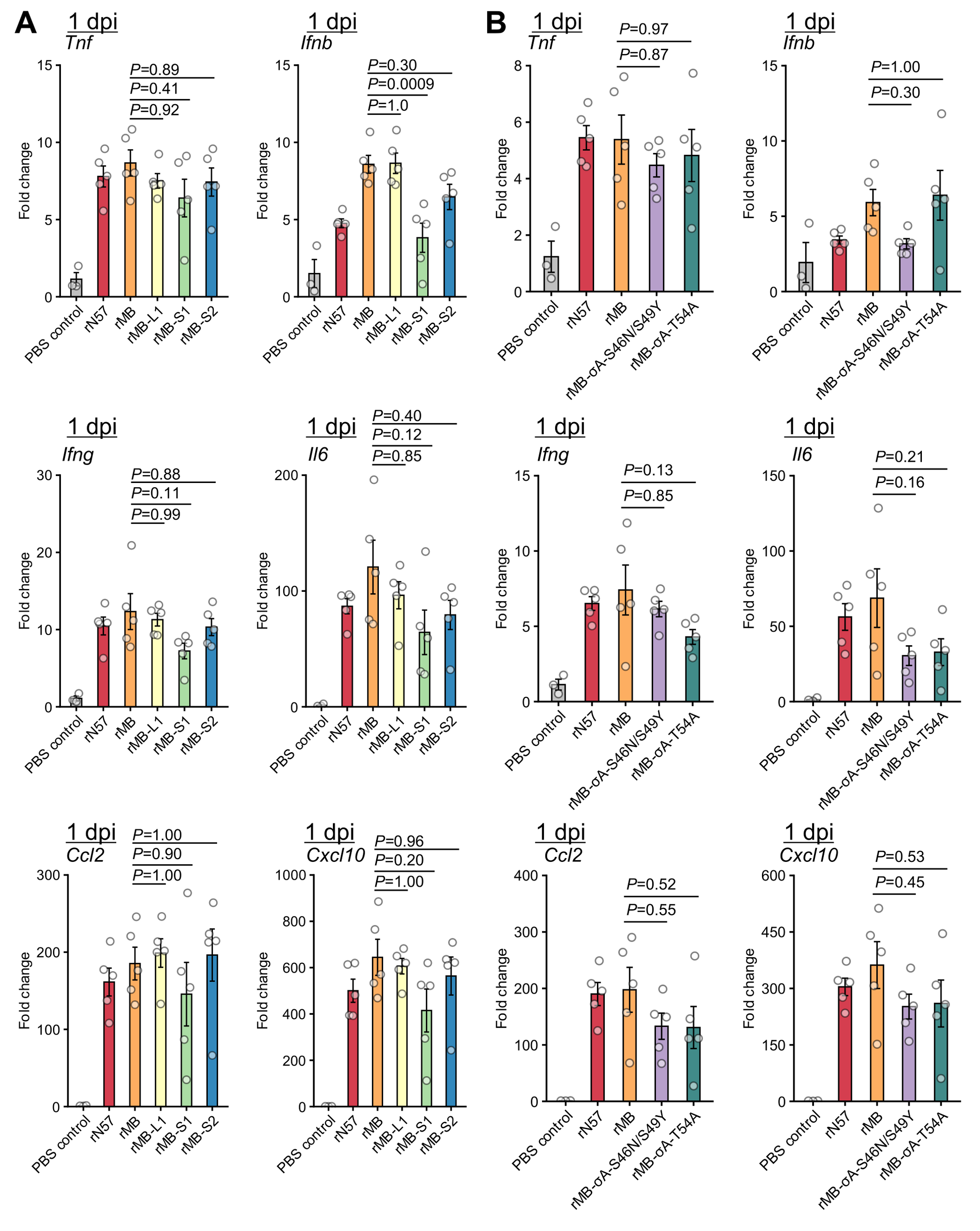

Supplement: S3 Fig — (A) mRNA levels of Tnf, Ifnb, Ifng, Il6, Ccl2, and Cxcl10 in lung tissue of mice infected with rN57, rMB, rMB-L1, rMB-S1, or rMB-S2 at 1 dpi. (B) mRNA levels of Tnf, Ifnb, Ifng, Il6, Ccl2, and Cxcl10 in lung tissue of mice infected with rN57, rMB, rMB-σA-S46N/S49Y, or rMB-σA-T54A at 1 dpi. Data were normalized to mouse β-actin (Actb). Each value represents the mean ± SEM for each group (n = 5). Each dot represents an individual mouse. dpi, days post infection; PBS, phosphate-buffered saline; rMB, recombinant MB; rN57, recombinant N57. (TIF) [file ppat.1014252.s013.tif]

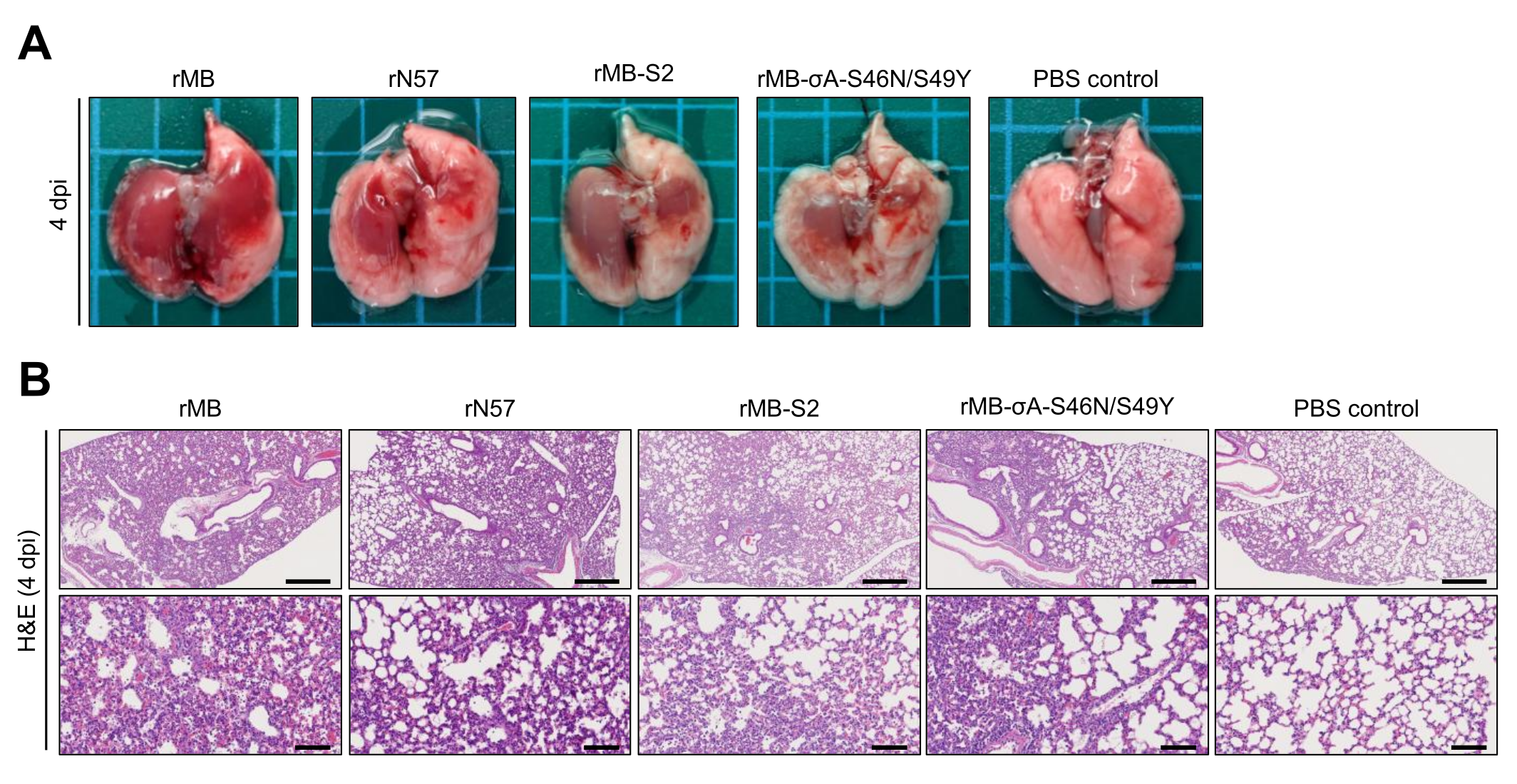

Supplement: S4 Fig — BALB/c mice (4 weeks old) were infected intranasally with 1 × 10^6^ PFU of rN57, rMB, rMB-S2, or rMB-σA-S46N/S49Y or with PBS (control). Lungs were harvested at 4 dpi for histological examination. (A) Macroscopic images of the infected lungs. (B) Histopathological images of infected lung tissue following sectioning and H&E staining. Higher magnifications of the sections in the first images (scale bars = 500 μm) are shown in the second images (scale bars = 100 μm). dpi, day post infection; H&E, hematoxylin and eosin; rMB, recombinant MB; rN57, recombinant N57. (TIF) [file ppat.1014252.s014.tif]

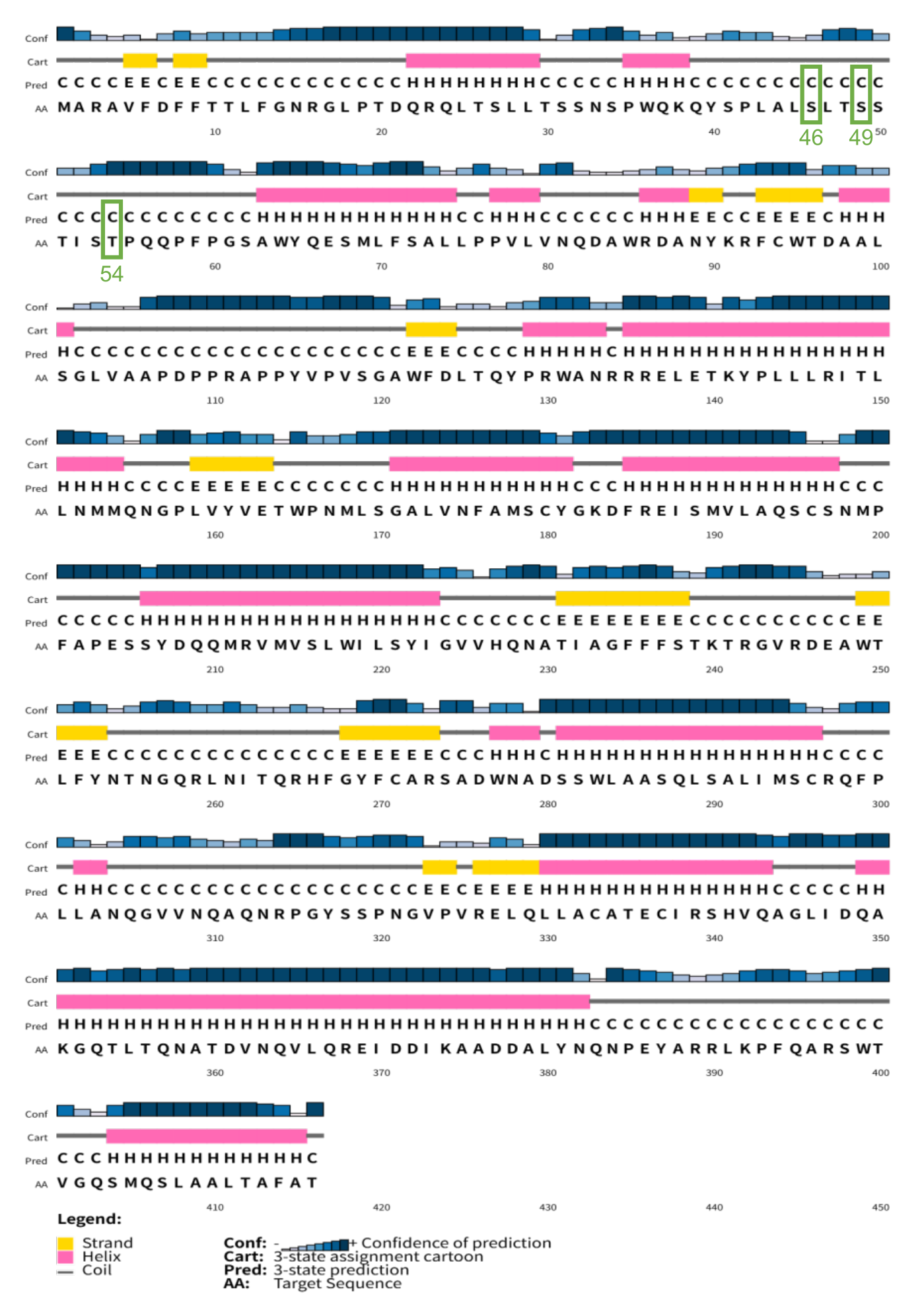

Supplement: S5 Fig — Pink, helices; yellow, β strands; gray, coils. The positions of σA Ser-46, Ser-49, and Thr-54 in the coil are highlighted within green squares. (TIF) [file ppat.1014252.s015.tif]
